# Does robotic-assisted esophagectomy improve outcomes compared to other techniques? An NCDB analysis of access and disparities

**DOI:** 10.1007/s00464-026-12614-x

**Published:** 2026-02-09

**Authors:** Claire Perez, Vikram Krishna, Lucas Weiser, Allen Razavi, Kellie Knabe, Sevannah G. Soukiasian, Raffaele Rocco, Philicia Moonsamy, Harmik J. Soukiasian, Andrew R. Brownlee

**Affiliations:** Division of Thoracic Surgery, Department of Surgery, Cedars-Sinai Medical Center, 8700 Beverly Blvd., Los Angeles, CA 90048 USA

**Keywords:** Esophagectomy, Disparities, Robotic-assisted surgery

## Abstract

**Objective:**

Surgery remains the gold standard for non-metastatic esophageal cancer. Oncologic resection is considered adequate when 15 regional lymph nodes are sampled and specimen margins are negative. We hypothesize that racial and regional disparities exist in who receives an adequate oncologic resection.

**Methods:**

The National Cancer Database (NCDB) was queried from 2010 to 2021 for patients who underwent esophagectomy for cancer. Exclusion criteria included stage IV disease and incomplete data. Adequate resection was defined as ≥ 15 lymph nodes removed and negative margins. A multivariable regression model identified factors associated with adequate resection, and survival was assessed using Kaplan–Meier curves.

**Results:**

11,451 patients were included. Of these, 5153 (45.0%) had an adequate oncologic resection. Black patients had increased odds of an inadequate resection compared to white patients (OR 1.490, 95%CI 1.227–1.809, *p* < 0.01). Patients treated at community or comprehensive cancer programs had higher odds of inadequate resection than those treated at academic programs. Medicaid patients had higher odds of an inadequate resection compared to those with private insurance (OR 1.397, 95%CI 1.172–1.664, *p* = < 0.01), while a minimally invasive esophagectomy (MIE) had 24.0% decreased odds of inadequate resection, and robotic-assisted esophagectomy (RAMIE) had 35.4% decreased odds compared to open surgery (95%CI 0.695–0.830, *p* < 0.01; 95%CI 0.567–0.735, *p* < 0.01). Controlling for stage, 5-year survival was higher for patients with an adequate resection. Resection adequacy improved from 38.5% in 2010 to 60.1% in 2021, with increases in MIE and RAMIE.

**Conclusion:**

Disparities persist in who receives adequate resection for esophageal cancer, though overall resection adequacy has improved, these findings should be interpreted in the context of evolving practice patterns.

**Supplementary Information:**

The online version contains supplementary material available at 10.1007/s00464-026-12614-x.

Esophageal cancer ranks as the sixth leading cause of cancer-related death worldwide and the overall prevalence is increasing [1]. For local or locally advanced disease, gold standard treatment often involves resection, with neoadjuvant therapy recommended based on overall T-stage and local lymph node metastasis [2]. In these patients, esophagectomy is performed with a curative intent and requires extensive pre-operative optimization and careful patient selection given its high morbidity and mortality [3].

As experience with minimally invasive techniques grows, an increasing number of esophagectomies are being performed as a minimally invasive esophagectomy (MIE) or a robotic-assisted minimally invasive esophagectomy (RAMIE) [4]. In some studies, less invasive techniques, particularly RAMIE, have demonstrated superiority in lymph node harvest during esophagectomy [5–8]. Lymph node status has been shown to be a key prognostic factor and an independent predictor for survival [9, 10]. Some evidence suggests that a more extensive lymphadenectomy may improve survival, even in the context of node negative disease [11]. As such, National Comprehensive Cancer Network (NCCN) guidelines dictate a minimum of 15 lymph nodes be removed during an esophagectomy for adequate staging and subsequent treatment [12].

Despite this recommendation, there still is not a standardized approach to lymphadenectomy for esophageal cancer [13]. As a potential consequence, evidence has shown that less than 50% of patients who underwent an esophagectomy between 2004 and 2015 received an adequate lymphadenectomy [14]. Considering the margin status on the primary specimen and adequacy of lymphadenectomy, it is likely that even fewer patients received an overall adequate oncologic resection. Furthermore, given the widespread racial and socioeconomic disparities in healthcare in the US [15], certain populations may be more or less likely to receive adequate treatment for esophageal cancer.

Access to high-quality surgical care, including minimally invasive techniques like MIE and RAMIE, is unevenly distributed. Racial, socioeconomic, and insurance disparities affect both the likelihood of surgery and the quality of oncologic resection [16, 17]. This study examines national patterns in adequacy of oncologic resection using the NCDB, focusing on surgical approach, hospital volume, and patient demographics including access disparities.

## Methods

The National Cancer Database (NCDB) is a national oncologic database co-sponsored by American College of Surgeons Commission on Cancer and the American Cancer Society. It includes oncologic data from over 1500 cancer programs and includes all 50 states and Puerto Rico [18]. This database was queried for all patients with esophageal cancer from 2010 to 2021. Patients were excluded if they did not have surgery or underwent a partial esophagectomy, had Stage 4 or their procedure was classified as palliative, had unknown staging, lymph node resection, or margin status, and those with incomplete information including follow-up data.

Adequate oncologic resection was determined as patients who had both negative margins on final pathology and ≥ 15 lymph nodes resected. The primary outcome measure was the rate of adequate oncologic resection as a factor of demographic and cancer-related variables. Secondary outcomes included unplanned readmission rate, 30- and 90-day mortality, and 5-year overall survival based on adequacy of oncologic resection. Centers were classified as high-volume if they performed over 20 cases annually, mid-volume if they conducted between 7 and 20 cases per year, and low-volume if they handled fewer than 7 cases annually [19].

Baseline patient and facility characteristics were reported as means ± standard deviation or median with interquartile range for continuous variables and as absolute values with percentages for categorical variables. Factors analyzed were year of diagnosis, patient factors, facility factors, disease factors, and post-operative factors. Patient factors included age, sex, race, insurance, and Charlson Deyo score. Facility factors included facility type, geographic location, general population of the city in which the facility is located, local high school equivalency and income quartiles based on zip code of the facility. Disease factors included pathologic stage, timing of radiation therapy, timing of chemotherapy and surgical approach. Post-operative factors included readmission and 30- and 90-day readmission.

A multivariable analysis was constructed for the adjusted comparison of adequate and inadequate oncologic resection. All patient, facility, disease, and post-operative factors were included in the analysis. To explore the potential influence of temporal changes in practice, a subgroup analysis was performed limited to patients treated from 2017 to 2021. Within this subgroup, rates of adequate oncologic resection were compared across surgical approaches and key demographic variables. Unadjusted survival was evaluated with the Kaplan–Meier method and stratified by stage including Stage 0 – Stage 3. Right censoring was performed 5 years after esophagectomy, and patients who did not reach these follow-up times were censored on the last follow-up date. Tests were two-tailed with an alpha level of 0.05. All statistical analyses were performed using SAS 9.4 (SAS Institute, Cary, North Carolina).

## Results

**Fig. 1:**
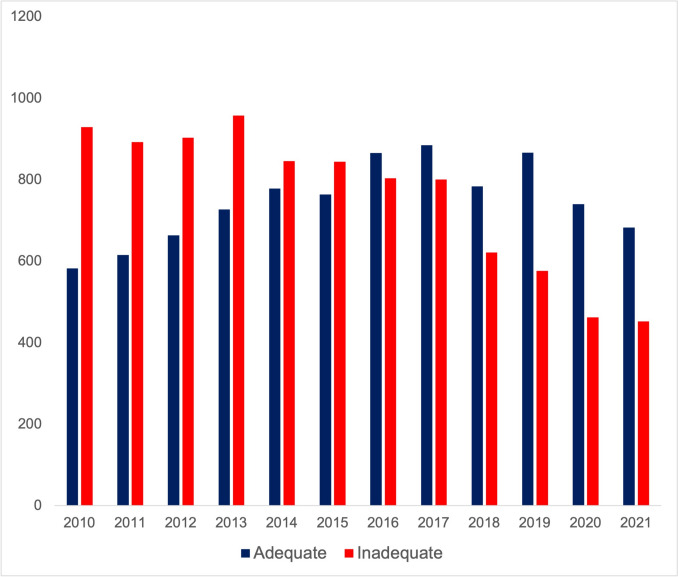
The number of inadequate and adequate resections over the course of the years

After exclusion, 11,451 patients were included in the analysis. A total of 6298 (55.0%) cases had inadequate oncologic resection with 5960 (52.0%) patients having fewer than 15 lymph nodes examined and 752 (6.6%) patients having a positive margin. Prior to exclusion of patients with missing survival data, there were 5153 (45.0%) patients. Between 2015 and 2016, there was a shift from less than 50% being deemed adequate to more than 50% being considered adequate (Fig. 1).

The two groups were comparable in terms of average age and sex distribution, with a Median age of 64 years and over 80% of patients being male (*p* = 0.52; *p* = 0.85). More than 90% of patients in both groups were White; however, 3.7% of the adequate resection group were Black compared to 5.6% in the inadequate group (*p* = < 0.01). Patients with inadequate resections were less likely to have private insurance or Medicare and more likely to be uninsured or covered by Medicaid compared to those with adequate resections (*p* < 0.01). The Charlson-Deyo comorbidity scores did not differ significantly between groups (*p* = 0.73; Table 1).

**Table 1:** Baseline characteristics of patients

Patient factors	Adequate *N* = 5153 (%)	Inadequate *N* = 6298 (%)	p-value
Age	64 (57–70)	64 (57–70)	0.52
Sex			
Male	4223 (82.0)	5170 (82.1)	0.85
Female	930 (18.1)	1128 (17.9)	
Race			
White	4770 (92.6)	5756 (91.4)	
Asian	104 (2.0)	100 (1.6)	< 0.01
Black	189 (3.7)	353 (5.6)	
Native American	12 (0.23)	20 (0.32)	
Pacific Islander	5 (0.10)	7 (0.11)	
Other	73 (1.4)	62 (0.98)	
Insurance			
Private insurance	2322 (25.1)	2629 (41.7)	< 0.01
Medicaid	266 (5.2)	432 (6.9)	
Medicare	2337 (45.4)	2897 (46.0)	
Other government insurance	156 (3.1)	196 (3.1)	
Not insured	72 (1.4)	144 (2.3)	
Charlson deyo score			
0	3527 (68.5)	4321 (68.6)	0.73
1	1208 (23.4)	1477 (23.5)	
2	298 (5.8)	340 (5.4)	
≥ 3	120 (2.3)	160 (2.5)	

Of those who had an adequate resection, 65.6% were performed at an academic or research program compared to 47.9% of those with an inadequate resection. Over 30% of patients in the inadequate group had their procedure at a comprehensive community cancer program compared to only 18.8% in the adequate group (*p* < 0.01). A higher percentage of adequate resections were performed in the south central and eastern US while a higher percentage of inadequate resections were done in the north and south-central US (*p* < 0.01).

Among the inadequate resection group, 18.8% of procedures were performed in areas with the highest quartile of local high school equivalency and 34.8% in regions with the highest income quartile. In comparison, the adequate resection group had 16.1% and 39.6% of procedures in these respective locations (*p* = < 0.01; *p* < 0.01. Table 2).

**Table 2:** Facility factors for the cohort

Facility factors	Adequate (%)	Inadequate (%)	*p*-value
Type			
Academic/research program	3331 (65.6)	2986 (47.9)	< 0.01
Community cancer program	44 (0.87)	136 (2.2)	
Comprehensive community cancer program	953 (18.8)	1926 (30.9)	
Integrated network cancer program	750 (14.8)	1186 (19.0)	
Case volume			< 0.01
High	853 (67.1)	418 (32.9)	
Middle	2189 (53.7)	1890 (46.3)	
Low	2111 (34.6)	3990 (65.4)	
Geographic location			
Western US	1048 (20.3)	1327 (21.1)	< 0.01
Eastern US	1465 (28.4)	1335 (21.2)	
North Central US	1222 (23.7)	1783 (28.3)	
South Central US	1343 (26.1)	1789 (28.4)	
City population			
Metropolitan	4305 (83.5)	5094 (80.9)	< 0.01
Urban	769 (14.9)	1079 (17.1)	
Rural	79 (1.5)	125 (2.0)	
Local highschool equivalency			
15.3% +	827 (16.1)	1184 (18.8)	< 0.01
9.1%—15.2%	1522 (29.6)	1822 (29.0)	
5.0%—9.0%	1670 (32.5)	1991 (31.6)	
< 5.0%	1128 (21.9)	1296 (20.6)	
Local income quartiles			
$74,063 +	2033 (39.6)	2185 (34.8)	< 0.01
$57,856-$74,062	1270 (24.7)	1597 (25.4)	
$46,277-$57,856	1128 (22.0)	1514 (24.1)	
< $46,277	708 (13.8)	984 (15.7)	

*US* United States

In both groups, the highest percentage of patients had Stage 3 disease (Adequate: 39.8%, Inadequate: 37.3%; *p* < 0.01). Of those with an adequate resection, 30.6% had no radiation and 64.5% had neoadjuvant therapy compared to 27.6% and 67.2% in the inadequate group, respectively (*p* = < 0.01). A higher percentage of those in the inadequate group had neoadjuvant chemotherapy (68.1%) compared to the adequate group (64.9%; *p* < 0.01).

Overall, more patients who had a MIE and a RAMIE had an adequate resection (*p* < 0.01; Table 3). There was no significance difference between groups regarding unplanned readmission rates, though 5.2% and 11.1% of inadequately resected patients died within 30 and 90 days, respectively. Only 3.8% and 7.9% died within 30 and 90 days after adequate resection (*p* < 0.01; *p* < 0.01; Table 4).

**Table 3:** Disease factors for the cohort

Disease factors	Adequate (%)	Inadequate (%)	p-value
Pathologic stage			
Stage 0	56 (1.1)	98 (1.6)	< 0.01
Stage 1	1067 (20.7)	1172 (18.6)	
Stage 2	1529 (29.7)	1960 (31.1)	
Stage 3	2052 (39.8)	2347 (37.3)	
Radiation therapy			
None	1575 (30.6)	1738 (27.6)	< 0.01
Adjuvant	229 (4.4)	299 (4.8)	
Neoadjuvant	3322 (64.5)	4231 (67.2)	
Both	27 (0.52)	30 (0.48)	
Chemotherapy			
None	1237 (24.0)	1409 (22.4)	< 0.01
Adjuvant	278 (5.4)	347 (5.5)	
Neoadjuvant	3344 (64.9)	4290 (68.1)	
Both	294 (5.7)	252 (4.0)	
Number of lymph nodes taken	21 (17–26)	9 (5–12)	< 0.01
Margin status			
Residual tumor	0 (0)	314 (5.0)	< 0.01
Microscopic residual tumor	0 (0)	406 (6.5)	
Macroscopic residual tumor	0 (0)	32 (0.51)	
No residual tumor	0 (0)	5524 (87.7)	
Surgical approach			
Open	3033 (58.9)	4280 (68.0)	< 0.01
Minimally invasive	1509 (29.3)	1485 (23.6)	
Robotic assisted	611 (11.9)	533 (8.5)

**Table 4:** Post-operative factors for the cohort

Post-operative factors	Adequate	Inadequate	p-value
Readmission			
Not readmitted	4749 (92.2)	5754 (91.4)	0.31
Planned readmission	55 (1.1)	87 (1.4)	
Unplanned readmission	327 (6.3)	419 (6.7)	
Short-term mortality			
30-day mortality	231 (3.8)	371 (5.2)	< 0.01
90-day mortality	484 (7.9)	795 (11.1)	< 0.01

Of those patients with inadequate oncologic resections, 314 (5.0%) patients had residual tumor, 406 (6.5%) patients had microscopic residual tumor, and 32 (0.51%) had macroscopic residual tumor (Table 3).

Hospital volume also differed significantly by surgical approach (p < 0.01). High-volume centers performed a greater proportion of RAMIE cases (16.5%) compared to minimally invasive (11.9%) and open (9.9%) surgeries. Conversely, low-volume centers accounted for the majority of open (56.8%) and minimally invasive (46.0%) cases, and nearly half of RAMIE cases (47.3%) (Supplemental Table 1). Regarding pathologic staging by surgical approach, there were significant differences observed (*p* < 0.01). MIE and RAMIE were more frequently performed in patients with earlier stage disease (Stage 0 and Stage 1), comprising 31.2% and 28.6% for MIE, and 9.1% and 9.9% for RAMIE, respectively, compared to open surgery patients who had a higher proportion of Stage 2 and Stage 3 disease (64.1% and 63.5%) (Supplemental Table 2).

In our cohort, 30-day and 90-day mortality varied significantly by surgical approach. Patients undergoing open esophagectomy had the highest observed 30-day mortality at 4.3%, compared to 2.7% with minimally invasive esophagectomy (MIE) and 3.6% with robotic-assisted minimally invasive esophagectomy (RAMIE) (*p* = < 0.01). Similarly, 90-day mortality was highest in the open surgery group (8.8%), followed by RAMIE (7.5%) and MIE (6.4%) (*p* = < 0.01) (Supplemental Table 3).

Given the observed temporal shift in resection adequacy, a subgroup analysis was performed among patients treated from 2017 to 2021 to evaluate whether differences in oncologic resection adequacy persisted in the contemporary era. Within this subgroup, rates of adequate oncologic resection remained higher among patients undergoing minimally invasive and robotic-assisted esophagectomy compared with open surgery (53.2% vs. 47.3%, *p* = 0.02, Supplemental Table 4).

On multivariable analysis, significant variables within patient factors included Black race when compared to White and Medicaid and no insurance compared to private insurance. Black patients had 49.0% higher odds of receiving an inadequate resection compared to White patients (*p* < 0.01). Medicaid patients had 39.7% increased odds of receiving an inadequate resection than those with private insurance (*p* = < 0.01). All evaluated facility types had greater odds of inadequate oncologic resection compared to academic/research programs (*p* < 0.01, Table 5). Similarly, all regions had higher odds of inadequate resection compared to the Western US but with only North Central US achieving significance (OR 1.211, 95%CI 1.078–1.361, *p* = < 0.01). Low-volume centers and mid-volume centers had higher odds of inadequate resections compared to high-volume centers (OR 1.590 [1.384–1.826], *p* < 0.01 and OR 2.989 [2.588–3.452], *p* < 0.01, respectively). Patients who underwent surgery at a facility where the local high school equivalence is in the 3rd quartile had 17% less odds of receiving an inadequate resection compared to those in the 4th quartile (OR 0.830, 95%CI 0.734–0.938, *p* = < 0.01).

**Table 5:** Multivariable analysis predicting the likelihood for inadequate resection

Variable	Odds Ratio (95% CI)	p-value
Sex		
Male	1.00	
Female	0.980 (0.885–1.084)	0.70
Race		
White	1.00	
Asian	0.952 (0.710–1.277)	0.74
Black	1.490 (1.227–1.809)	** < 0.01**
Native American	1.476 (0.701–3.108)	0.31
Pacific Islander	1.388 (0.428–4.505)	0.56
Other	0.774 (0.466–1.284)	0.32
Insurance		
Private insurance	1.00	
Medicaid	1.397 (1.172–1.664)	** < 0.01**
Medicare	1.061 (0.977–1.152)	0.16
Other government insurance	1.000 (0.744–1.344)	1.00
Not insured	1.630 (1.205–2.207)	** < 0.01**
Charlson deyo score		
0	1.00	
1	0.965 (0.880–1.058)	0.44
2	0.873 (0.737–1.033)	0.11
≥ 3	0.934 (0.726–1.201)	0.59
Facility Type		
Academic/Research program	1.00	
Community cancer program	3.231 (2.273–4.594)	** < 0.01**
Comprehensive community cancer program	2.100 (1.906–2.313)	** < 0.01**
Integrated network cancer program	1.719 (1.543–1.915)	** < 0.01**
Case volume		
High	1.00	
Middle	1.590 (1.384–1.826)	** < 0.01**
Low	2.989 (2.588–3.452)	** < 0.01**
Geographic location		
Western US	1.00	
Eastern US	0.906 (0.806–1.018)	0.10
North Central US	1.211 (1.078–1.361)	** < 0.01**
South Central US	1.078 (0.962–1.208)	0.20
Highschool equivalency		
15.3% +	1.00	
9.1%—15.2%	0.830 (0.734–0.938)	** < 0.01**
5.0%—9.0%	0.877 (0.769–1.000)	0.05
< 5.0%	0.919 (0.789–1.071)	0.28
Income quartiles		
$74,063 +	1.00	
$57,856-$74,062	1.071 (0.963–1.191)	0.21
$46,277-$57,856	1.123 (0.998–1.264)	0.05
< $46,277	1.059 (0.914–1.226)	0.45
Pathologic stage		
Stage 0	1.00	
Stage 1	0.581 (0.409–0.827)	** < 0.01**
Stage 2	0.603 (0.421–0.863)	** < 0.01**
Stage 3	0.548 (0.382–0.784)	** < 0.01**
Radiation therapy		
None	1.00	
Adjuvant	1.248 (0.979–1.590)	0.07
Neoadjuvant	1.224 (1.047–1.430)	** < 0.01**
Both	1.104 (0.620–1.966)	0.74
Chemotherapy		
None	1.00	
Adjuvant	0.899 (0.713–1.134)	0.37
Neoadjuvant	1.021 (0.858–1.215)	0.82
Both	0.689 (0.543–0.873)	** < 0.01**
Approach		
Open	1.00	
Minimally invasive	0.760 (0.695–0.830)	** < 0.01**
Robotic assisted	0.646 (0.567–0.735)	** < 0.01**

Bold data indicates significant *p*-value

Of the disease-related and post-operative factors, significant variables included stage 2 and 1 compared to stage 0, adjuvant and neoadjuvant radiation therapy compared to none, combined adjuvant and neoadjuvant chemotherapy compared to none, MIE and RAMIE compared to open esophagectomy and 90-day mortality. There is a general trend toward decreased odds of inadequate oncologic resection as stage increases (Table 5). Those with neoadjuvant radiation had 22.4% increased odds of inadequate oncologic resection compared to those who received no radiation therapy (OR 1.224, 95%CI 1.047–1.430, *p* = 0.01). Patients who received neoadjuvant and adjuvant chemotherapy had 31.1% higher odds of inadequate resections (*p* = < 0.01). Those that underwent MIE had 24.0% less odds and RAMIE had 35.4%% less odds of inadequate oncologic resection compared to an open esophagectomy (*p* < 0.01, *p* < 0.01). Patients who had an inadequate oncologic resection had a significantly higher risk of mortality, with a hazard ratio of 1.582 (95%CI 1.300–1.926, *p* < 0.01) at 30 days and 1.639 (95%CI 1.427–1.883, *p* < 0.01) at 90 days compared to those with an adequate resection.

**Fig. 2:**
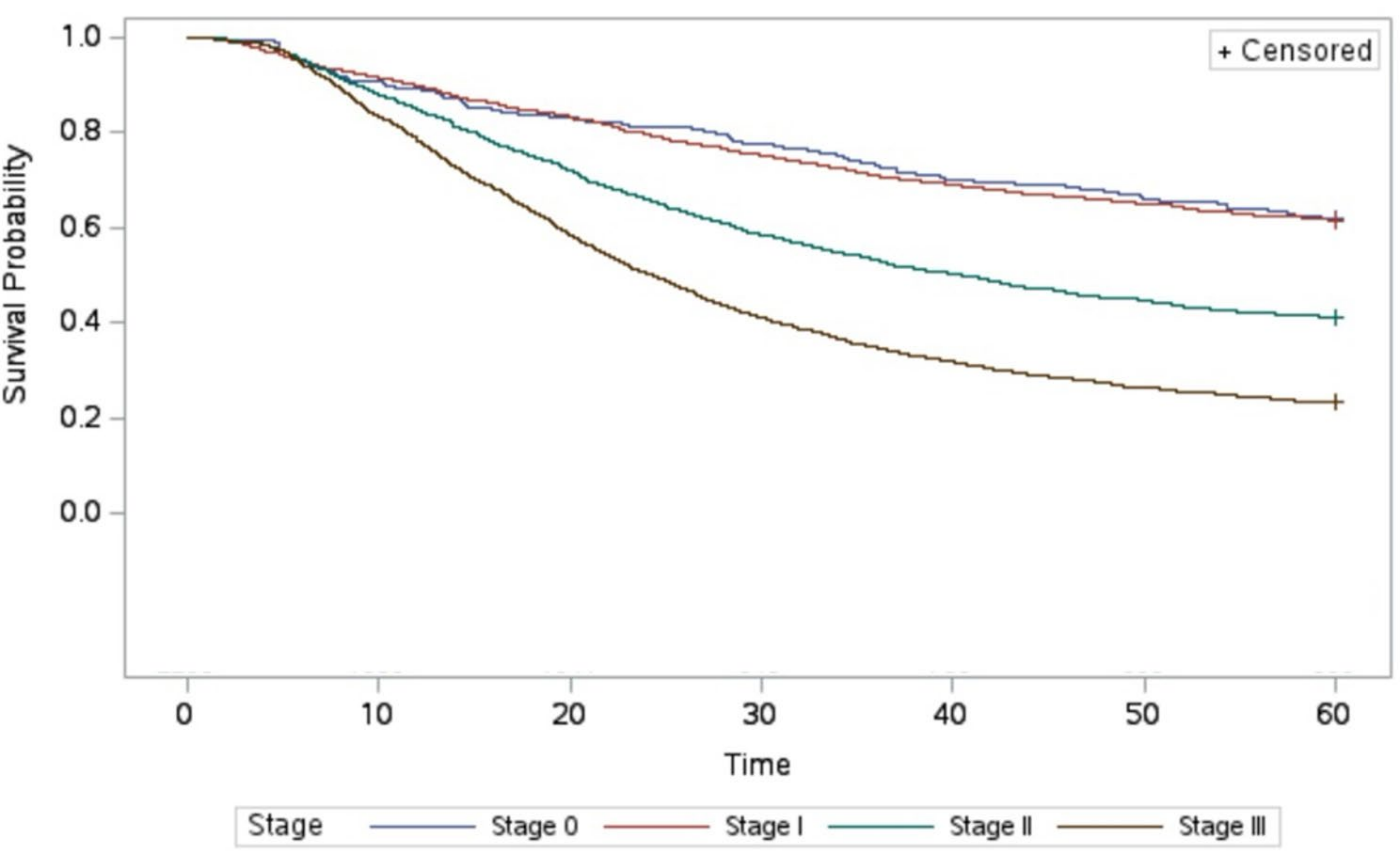
Five-year survival adequate resection stratified by pathologic stage

**Fig. 3:**
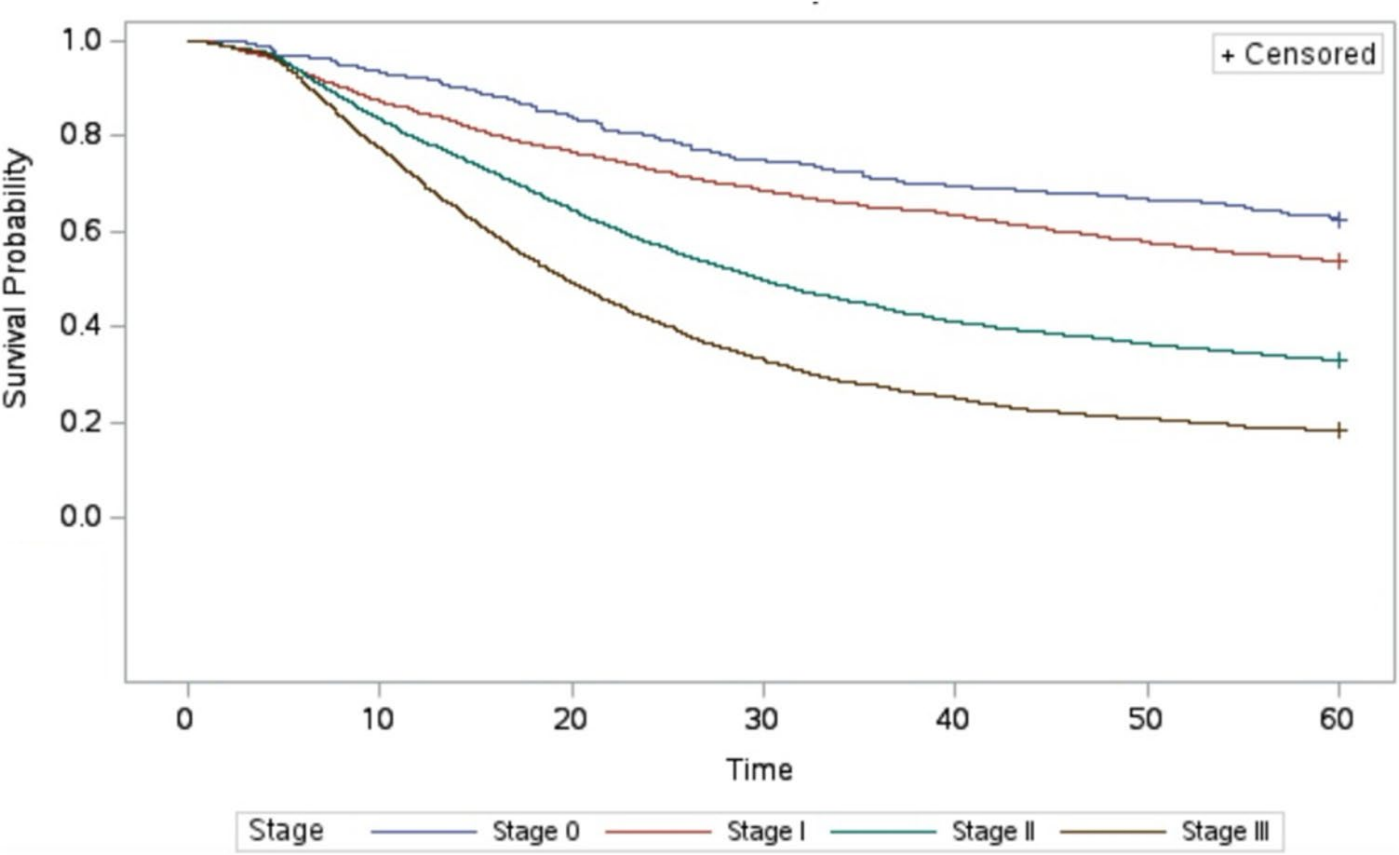
Five-year survival after inadequate resection stratified by pathologic stage

Overall unadjusted 5-year survival for Stage 0, 1, 2, and 3 patients after adequate oncologic resection was 62.1, 61.45, 41.02, and 23.3%. For those that underwent inadequate oncologic resection, 5-year survival was lower at all stages besides Stage 0 with survival rates by stage of 62.47, 53.82, 33.06, and 18.37%, respectively (*p* < 0.01; Figs. 2, 3).

## Discussion

In this contemporary analysis of the NCDB esophageal cancer patients, it is demonstrated that significant disparities exist impacting the likelihood of receiving an adequate oncologic resection. Furthermore, it highlights that a substantial proportion of patients overall do not undergo adequate oncologic resections, with this disparity primarily attributed to the insufficient number of lymph nodes removed. Although the frequency of adequate resections has been increasing annually, as of 2021, 39.9% of patients still did not receive an adequate oncologic resection. This finding is corroborated by the fact that the rates of inadequate lymphadenectomy and positive margins observed in this study align with those reported in previous literature [20, 21].

**Fig. 4:**
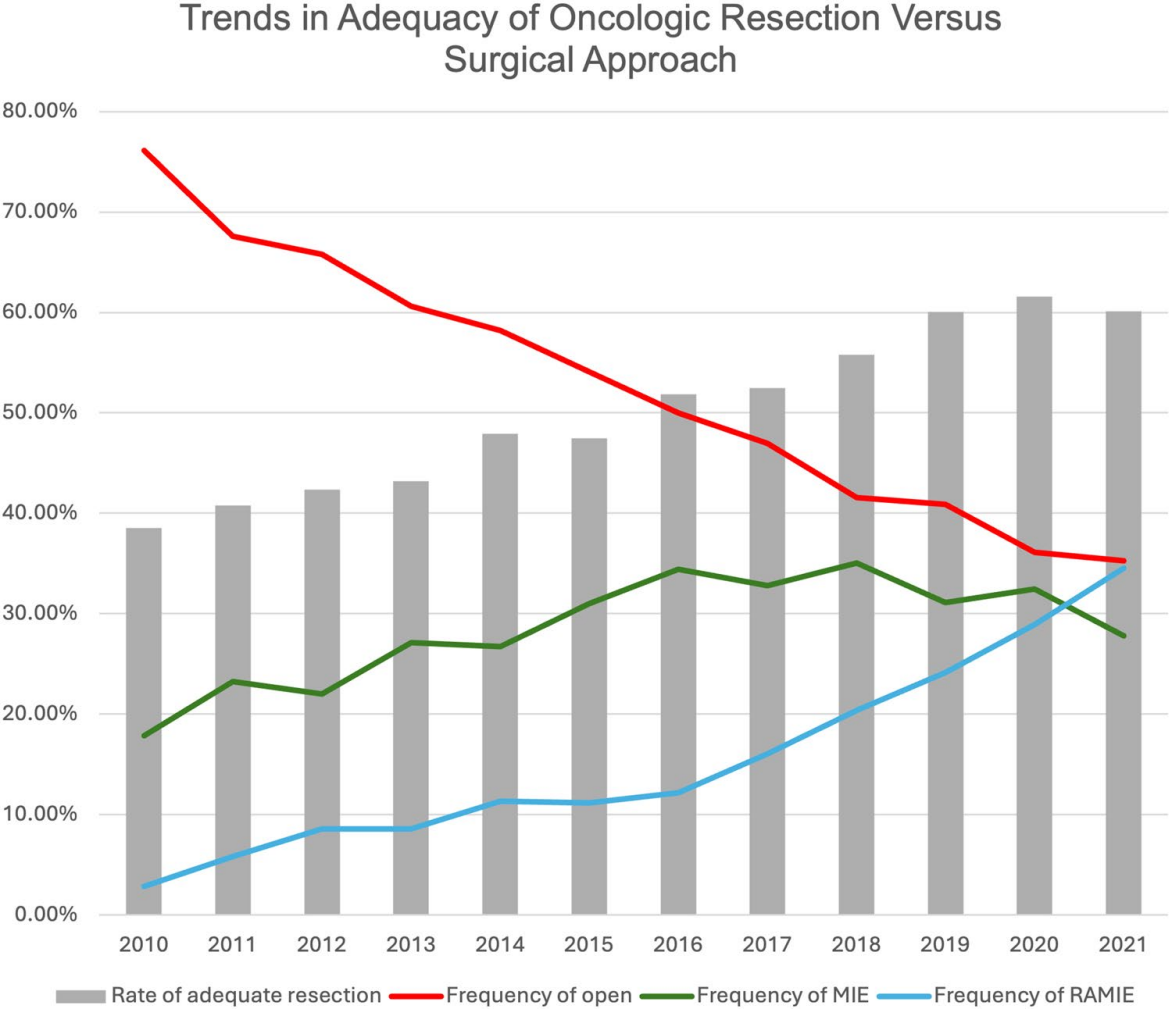
Trends in adequacy of oncologic resection versus surgical approach over the course of the years

During the study period, the use of both MIE and RAMIE increased while the frequency of open esophagectomy declined (Fig. 4). Notably, the adoption of robotic-assisted esophagectomy paralleled increases in the proportion of adequate resections, suggesting a potential association between minimally invasive approaches and thorough lymphadenectomy. On multivariable analysis, patients who underwent RAMIE or MIE had higher odds of adequate resection compared with open surgery. These observations are likely influenced by multiple factors, including evolving surgical practices, patient selection, and institutional patterns.

Notably, our analysis of case volume revealed that centers with mid-volume and low-volume esophagectomy practices had significantly higher odds of inadequate resections compared to high-volume centers (OR 1.590 [1.384–1.826], *p* < 0.01 and OR 2.989 [2.588–3.452], *p* < 0.01, respectively). These findings suggest that institutional experience and practice patterns may influence resection adequacy. While RAMIE was performed across centers of varying volume, a substantial proportion occurred at mid- and low-volume centers (Supplemental Table 2), indicating that surgical approach alone does not fully account for differences in oncologic outcomes.

As with many other diseases, evidence indicates that significant racial disparities exist in the incidence and outcomes of esophageal cancer. Black patients in particular seem to have a much higher odds of presenting with later stage disease [22] and a lower likelihood of being offered surgery [23]; non-white patients overall seem to have significantly lower median survival [24]. This analysis reveals that, in addition to the previously documented racial disparities in surgical access, the adequacy of oncologic resection may also play a significant role in exacerbating the broader racial disparities in esophageal cancer treatment.

Beyond racial disparity, there is also a significant difference among insurance types found in this study. The etiology of this disparity is likely multifactorial. It has been shown that lower socioeconomic status negatively impacts survival in esophageal cancer [20, 21] which is reflected with higher odds of receiving an inadequate oncologic resection for patients with Medicaid. It is possible that these patients are less likely to be treated at academic or research institutes compared to community programs which were much less likely to provide adequate resections. Given that academic faculty are more likely to be actively involved in research, thoracic surgical societies, and potentially committees on surgical guidelines, it is reasonable that these surgeons would be more likely to consistently sample ≥ 15 lymph nodes.

Interestingly, higher stage when compared to stage 0 resulted in higher odds of receiving an adequate oncologic resection. Patients with stage 0, or a complete pathologic response to neoadjuvant therapy, may be less likely to receive a thorough lymphadenectomy. Furthermore, these patients may have undergone extensive neoadjuvant therapy that could increase the overall difficulty of the surgery. This is consistent with the finding that patients who underwent neoadjuvant radiation therapy had an increased odds of an inadequate resection. Furthermore, higher stage patients having an adequate resection may be secondary to upstaging. Those that have a more thorough lymphadenectomy may be more likely to be upstaged after final pathology, particularly between stage 2 and stage 3. In addition, patients that had inadequate oncologic resection had higher rates of neoadjuvant chemotherapy, which may also explain the inadequacy of lymphadenectomy due to disease burden.

To further examine whether inadequate oncologic resection is primarily attributable to surgical approach or to disease-related factors, we performed a multivariable analysis adjusting for both. Our findings indicate that while advanced pathologic stage and absence of pre-operative therapy are associated with inadequate resection, the surgical approach remains an independent predictor. RAMIE and MIE were both associated with higher odds of adequacy compared to open surgery, even after controlling for tumor stage and treatment modality. These results suggest that both surgical technique and disease burden contribute to resection adequacy.

We also expanded our data presentation to include perioperative outcomes stratified by surgical approach. Specifically, 30-day and 90-day mortality differed significantly across operative types, with open surgery associated with the highest short-term mortality. RAMIE showed intermediate mortality rates, while MIE had the lowest. Importantly, patients who received inadequate oncologic resections had significantly higher 30- and 90-day mortality, irrespective of surgical approach. This suggests that inadequate resection may not only be a surrogate for technical quality but also directly contributes to worse early outcomes.

To further investigate whether the higher unadjusted mortality observed in the RAMIE cohort reflected the surgical approach or underlying case-mix differences, we performed a multivariable logistic regression adjusting for age, sex, comorbidity index, pathologic stage, systemic therapy, radiation, and geographic/urban factors. After adjustment, RAMIE was not independently associated with increased 30-day mortality (OR = 0.79, *p* = 0.17), indicating that the higher crude mortality rate was likely driven by case selection. Laparoscopic cases, by contrast, showed significantly lower adjusted mortality relative to open surgery (OR = 0.61, *p* < 0.01). These findings highlight that RAMIE can be performed safely in appropriately selected patients, and that the observed unadjusted mortality should not be interpreted as a limitation of the robotic approach itself.

The 30-day and 90-day mortality were significantly different between the two groups in that those who received inadequate resection had higher odds of 30-day and 90-day mortality. However, readmission rates and 30-day mortality were not significantly associated with adequate resection, though 90-day mortality was associated with adequate resection. This may be confounded by stage given higher stages are also associated with oncologic resection. It is feasible that higher stage patients have higher 90-day mortality. For all-cause mortality, regardless of stage, inadequate oncologic resection results in a significantly lower chance of survival. This finding carries more weight given that higher stages are also associated with adequate resections. Patients with adequate resections, despite having higher stage on average, have a higher rate of five-year overall survival.

While this study is descriptive, it identifies several potential avenues for intervention. Patterns observed in this study suggest that differences in institutional volume, surgical approach, and access to minimally invasive techniques are associated with variability in resection adequacy. Further research could explore referral patterns, surgeon training, and other factors that may contribute to these disparities.

This study has several limitations. The NCDB carries many innate limitations given its lack of granular data regarding specific types of esophagectomy, tumor size, types of perioperative therapies, length of stay, comorbidities, and other factors that could potentially confound the results. It also lacks granular operative data, such as operative time, blood loss, and type of complications. In particular, the absence of data on operating time and hospital length of stay restricts assessment of perioperative efficiency and recovery—two critical factors when comparing open versus minimally invasive approaches. It also does not include key clinical variables such as performance status, functional capacity, or frailty indices, all of which are important in surgical decision-making and could impact both the chosen approach and oncologic outcomes. Furthermore, 5-year survival is calculated as all cause and requires censoring due to lack of data. Staging is assumed pathologic in this study though the NCDB does not explicitly state the type of staging, rather provides pathologic staging if available. Given all patients in this cohort underwent a resection, it is reasonable to assume all staging is pathologic. Finally, the NCBD does not report margin size for resected specimens; therefore, the adequacy of resection cannot be assessed beyond the presence of macroscopic, microscopic, or residual tumor.

Despite these limitations, this study demonstrates a key finding—adequate oncologic resection is still not being performed consistently for esophageal cancer. Moreover, factors such as race, socioeconomic status, access to academic medical institutions, and availability of minimally invasive techniques significantly impact the likelihood of receiving an oncologic resection, with minority patients being notably less likely to undergo the procedure compared to their non-minority counterparts. Observed trends suggest that both disease characteristics and surgical practice patterns contribute to variability in outcomes. These findings emphasize the importance of further research to understand and address disparities in esophageal cancer care.

## Supplementary Information

Below is the link to the electronic supplementary material.

Supplementary file1 (DOCX 15 kb)
