# Supplementary material for: Does robotic-assisted esophagectomy improve outcomes compared to other techniques? An NCDB analysis of access and disparities
Source: Surg Endosc. 2026 Feb 9;40(4):3357–67. doi: 10.1007/s00464-026-12614-x (PMC13053566; doi:10.1007/s00464-026-12614-x)
Supplement: Supplementary file 1 — Supplementary file1 (DOCX 15 kb) [file 464_2026_12614_MOESM1_ESM.docx]

| Pathologic Staging by Surgical Approach | | | | | |
| --- | --- | --- | --- | --- | --- |
| Surgical Approach | Stage 0 | Stage 1 | Stage 2 | Stage 3 | *p*-value |
| Minimally Invasive | 48 (31.2) | 641 (28.6) | 889 (25.5) | 1143 (26.0) | <0.01 |
| Open | 92 (59.7) | 1376 (61.5) | 2237 (64.1) | 2795 (63.5) |  |
| Robotic-Assisted | 14 (9.1) | 222 (9.9) | 363 (10.4) | 461 (10.5) |  |

Table 1:

| Hospital Volume per Surgical Approach | | | | |
| --- | --- | --- | --- | --- |
| Volume | Open | Minimally Invasive | Robotic-Assisted | *p*-value |
| High | 726 (9.9) | 356 (11.9) | 189 (16.5) | <0.01 |
| Middle | 2433 (33.2) | 1233 (41.2) | 413 (36.1) |  |
| Low | 4154 (56.8) | 1405 (46.0) | 542 (47.3) |  |

Table 2:

| Mortality Rates by Surgical Approach | | | | |
| --- | --- | --- | --- | --- |
|  | Open | Minimally invasive | Robotic-assisted | *p*-value |
| 30 day mortality | 316 (4.3) | 80 (2.7) | 41 (3.6) | <0.01 |
| 90 day mortality | 641 (8.8) | 193 (6.4) | 84 (7.5) | <0.01 |

Table 3

| Adequacy of Resection after 2017 | | |  |
| --- | --- | --- | --- |
|  | Adequate | Inadequate | *p*-value |
| MIS (both laparoscopic and RAMIE) | 385 (53.2) | 339 (46.8) | 0.02 |
| Open | 355 (47.3) | 396 (52.7) |  |

Table 4:
